# Late-season biosynthesis of leaf fatty acids and *n*-alkanes of a mature beech (*Fagus sylvatica*) tree traced *via* ^13^CO_2_ pulse-chase labelling and compound-specific isotope analysis

**DOI:** 10.3389/fpls.2022.1029026

**Published:** 2023-01-06

**Authors:** Tatjana C. Speckert, Fanny Petibon, Guido L. B. Wiesenberg

**Affiliations:** Department of Geography, University of Zurich, Zurich, Switzerland

**Keywords:** cuticular leaf waxes, fatty acids, *n*-alkanes, *Fagus sylvatica*, ^13^CO_2_ labelling, compound-specific isotope analysis (CSIA)

## Abstract

Leaf cuticular waxes play an important role in reducing evapotranspiration *via* diffusion. However, the ability of mature trees to regulate the biosynthesis of waxes to changing conditions (e.g., drought, light exposition) remain an open question, especially during the late growing season. This holds also true for one of the most widely distributed trees in Central Europe, the European beech tree (*Fagus sylvatica L.*). In order to investigate the ongoing formation of wax constituents like alkanes and fatty acids, we conducted a ^13^CO_2_ pulse-chase labelling experiment on sun-exposed and shaded branches of a mature beech tree during the late summer 2018. The ^13^C-label was traced *via* compound-specific δ^13^C isotope analysis of *n*-alkanes and fatty acids to determine the *de-novo* biosynthesis within these compound classes. We did not observe a significant change in lipid concentrations during the late growing season, but we found higher *n*-alkane concentrations in sun-exposed compared to shaded leaves in August and September. The *n-*alkane and fatty acid composition showed ongoing modifications during the late growing season. Together with the uptake and following subsequent decrease of the ^13^C-label, this suggests ongoing *de-novo* biosynthesis, especially of fatty acids in European beech leaves. Moreover, there is a high variability in the ^13^C-label among individual branches and between sun-exposed and shaded leaves. At the same time, sun-exposed leaves invest more of the assimilated C into secondary metabolites such as lipids than shaded leaves. This indicates that the investigated mature beech tree could adjust its lipid production and composition in order to acclimate to changes in microclimates within the tree crown and during the investigated period.

## 1 Introduction

Climate scenarios predict substantial warming in Europe with an increase in summer temperature of 2-4°C (2081-2100, RCP4.5; Bussotti et al., 2015). Increasing temperature together with altered precipitation regimes and increased disturbance frequency is expected to decrease productivity and affect tree species distribution and community dynamics (McDowell et al., 2020). This may have negative effects on forest ecosystem services, including reduced carbon sequestration (McDowell et al., 2020). European beech (*F. sylvatica*), one of the most abundant trees in Central Europe (Blessing et al., 2015) and as such an ecological and economically important species, is known to be sensitive to drought, which can limit growth and competitiveness (Walthert et al., 2021). For such species with a wide distribution, there is great interest in how they can adapt to changing climatic conditions. To assess the sensitivity of *F. sylvatica* to future climate warming, numerous studies have investigated changes in foliar functional traits such as leaf size (Meier and Leuschner, 2008), bud burst or leaf senescence (Zohner and Renner, 2019) in response to environmental parameters. Another approach is to trace carbon allocation in beech trees with respect to environmental conditions using molecular proxies and compound specific-isotope analysis. One example is the combination of ^13^CO_2_ pulse labelling of tree saplings and chasing the ^13^C-abundance in different leaf compounds, such as starch, sugar, and amino acids (Blessing et al., 2015). As the leaf wax composition is sensitive to environmental conditions including exposure to radiation, heat, and droughts, leaf waxes were identified as a promising proxy to better understand tree response to future climate (Shepherd and Griffiths, 2006). However, only a few studies have looked at changes in leaf wax amount and composition of mature trees as a response to environmental variability during one growing season (Gülz and Müller, 1992; Lockheart et al., 1997; Sachse et al., 2009; Vega et al., 2020).

The cuticular wax layer consists of straight-chain aliphatic compounds (C_20_ - C_34_) derived from very long-chain fatty acids (FA) *via* the decarbonylation pathway (Jetter and Kunst, 2008). The cuticular wax layer is organized as epicuticular waxes located at the outermost leaf surface and intracuticular waxes embedded in the cutin biopolymer matrix. The epicuticular waxes interact with the environment and contribute to the protection of leaves, e.g., against ultraviolet light exposure and uncontrolled water loss *via* diffusion. Besides, due to their exposure at the leaf surface, cuticular long-chain FA and *n*-alkanes can be removed by wind or dust abrasion (Nelson et al., 2017) and transported over long distances followed by deposition into soils and sediments (Jansen and Wiesenberg, 2017). Therefore, their biosynthesis as well as their composition is expected to be actively adjusted in response to environmental conditions (Müller and Riederer, 2005; Shepherd and Griffiths, 2006). This might suggest a continuous renewal of wax components in tree leaves. Also Prasad and Gülz (1990) observed that the amount of leaf waxes and their composition change within one growing season with an increasing FA concentration in beech leaves towards the end of the growing season. This is in line with the dynamic wax modifications in leaves of common oak (*Quercus robur*; Gülz and Müller, 1992) and ivy (*Hedera helix* L.) with increasing leaf age (Hauke and Schreiber, 1998).

On top of the molecular composition, also compound-specific isotope variability was proven useful to trace carbon uptake and allocation in plant wax components (Heinrich et al., 2015). Sachse et al. (2009) showed strong seasonal variations in the natural hydrogen isotopic composition of leaf wax *n*-alkanes of beech trees. They observed a strong increase in *n*-C_27_ δ^2^H values (-140‰) in early October and a decrease (20‰) afterwards indicating a *de-novo* biosynthesis of *n*-alkanes during the entire growing season. In contrast, Kahmen et al. (2011) found that the δ^2^H values of alkanes of *Populus trichocarpa* are fixed in the early stage of leaf development and do not change during the entire life span of the leaf. This implies that the *n*-alkane δ^2^H values only record a short period of the environmental conditions during the entire leaf life span, which suggests a wax *n*-alkane synthesis only in the early stage of leaf development.

However, there is still little evidence for such renewal of important wax components such as long-chain FA and *n*-alkanes of broadleaf tree leaves during the growing season (Lockheart et al., 1997; Sachse et al., 2009). Additionally, the relationship between leaf wax cycling and the environment remains complex as leaf wax abundance and composition vary with leaf ontogeny (Nguyen-Tu et al., 2007), time of leaf wax synthesis (Sachse et al., 2009), and among plant species (Dodd and Poveda, 2003). In addition, leaf waxes vary within an individual tree depending on canopy position (Bahamonde et al., 2018; Vega et al., 2020). The abundance and stable isotope composition of leaf *n*-alkanes, for instance, varies between sun-exposed and shaded leaves within the same tree canopy (Lockheart et al., 1997; Bush and McInerney, 2013). This argues for the acclimation of leaf wax biosynthesis to micro-environmental differences, which affects the abundance, composition, and renewal rate of the leaf wax compounds.

In this study, we aimed to evaluate the late seasonal dynamic in leaf waxes of sun-exposed and shaded leaves of a ~200-year-old beech tree (*F. sylvatica*). We performed a ^13^CO_2_ pulse-chase labelling experiment to determine the *de-novo* biosynthesis of lipid compound classes. Within these compound classes we analyzed the variation in the concentration and composition of *n*-alkanes and FA in sun-exposed vs. shaded leaves during the late growing season 2018. We focused on the molecular and stable carbon (^13^C) isotope composition of leaf lipids, including *n*-alkanes and FA. We hypothesized that (i) leaf wax *n*-alkane concentration and chain length are higher in sun-exposed when compared to shaded leaves as an acclimation to light exposure, (ii) a higher variability in leaf wax *n*-alkane composition, concentration, and isotope composition is expected in sun-exposed leaves than in shaded leaves due to the higher variability in temperature and sun exposure and (iii) leaf wax biosynthesis continues until the late stages of the growing season.

## 2 Materials and methods

### 2.1 Site description

Field work was carried out between August and October 2018 on the campus of the University of Zurich, Switzerland [47°23’44’’N, 8°32’57’’E; 540 m *a.s.l.*]. The climate is characterized by a mean annual air temperature of 9.3°C and a mean annual precipitation of 1134 mm (MeteoSwiss, 2018a, accessed:2018). In the observation time period of the current study between August and October 2018, the average air temperature was 20.8°C (long-term average for 1981-2010: 18.0°C) and the cumulative precipitation was 113mm (long-term average for 1981-2010: 124 mm; MeteoSwiss, 2018b, accessed:2018).

### 2.2 ^13^CO_2_ pulse-chase labelling experiment and sample collection

A ^13^CO_2_ pulse-chase labelling experiment was conducted to trace carbon uptake and wax renewal during the late phase of the growing season (August to October 2018) starting with the ^13^CO_2_ pulse on 9^th^ August 2018. The maximum temperature on this sunny day was 27°C. A ~200-year-old *F. sylvatica* tree was selected in a group of five trees. The experiment was carried out at the sun-exposed south-west side of the tree on a very large branch, which had numerous well-developed terminal subbranches covering a ground area of ca. 50m^2^. In total, eight terminal branches were selected, which were located ca. 2 meters above the ground and had at least 60 leaves, each. Four of these subbranches were exposed to direct sunlight and four branches were located in a shaded position. In total, six branches (three sun-exposed and three shaded, respectively) were used for the ^13^CO_2_ labelling experiment. One additional branch in sun-exposed and another one in shaded position were used as unlabelled control branches to determine the natural isotope composition. The distance between all individual branches was at least 2 m from each other to avoid direct influence of the ^13^CO_2_ labelling experiment on the other branches.

**Figure 1:**
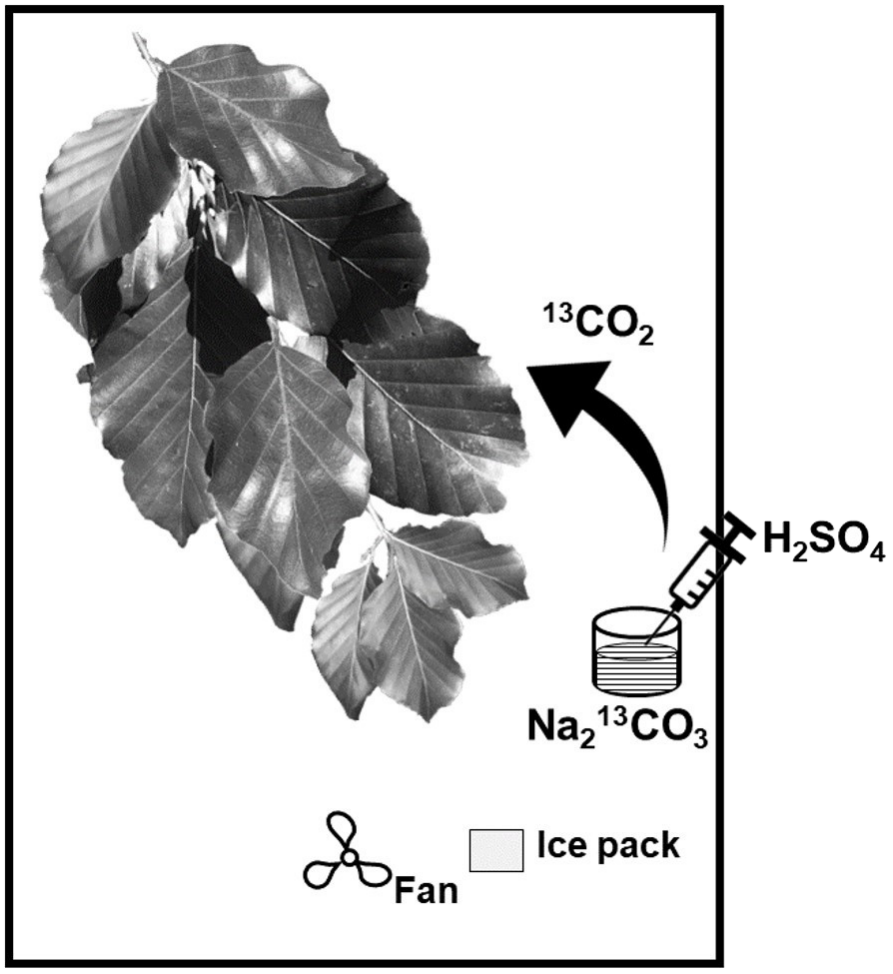
Schematic overview of the labelling chamber (modified after Kagawa et al., 2005).

Labelling chambers with a volume of 0.06m^3^ were placed and fixed on tripods around the branches that were chosen for the experiment. Each labelling chamber consisted of a wired frame built on a polypropylene bottom plate. Every frame was placed in transparent plastic foil that enabled >95% penetration of sunlight and UV radiation and was sealed air-tight around the top of the branches shortly before the beginning of the experiment. Battery-driven fans were placed in the chamber to homogenously distribute the air inside (Kagawa et al., 2005; Srivastava et al., 2018). Ice packs were placed in the chamber to lower air chamber temperature (**Figure 1**). Each labelling chamber contained a glass dish with an amount of 1g of the tracer (>99% Na_2_^13^CO_3_) dissolved in deionized water (Milli-Q quality). With this amount of tracer, we avoided an increase of the CO_2_ concentration inside the labelling chamber above 800 ppm at any time during the experiment. Thereby, the photosynthetic activity of the leaves and consequently CO_2_ uptake was maintained. The ^13^CO_2_ gas was released at the beginning of the experiment by injection of 10ml sulfuric acid (H_2_SO_4_, 10 atom-%) via a syringe directly through the plastic foil into the labelling solution after the fans were started and the bags were sealed. All branches were exposed to a ^13^CO_2_-enriched atmosphere for ~5 hours.

After 5 hours, the chambers were disassembled. Sun-exposed and shaded nodes containing at least three leaves, each, were collected at the topmost and terminal part of each branch immediately after the experiment (t0), as well as 4 and 7 days after labelling. Subsequently, leaves were collected weekly until 11^th^ October 2018, which resulted in 480 leaf samples during the chase-phase of the experiment. Immediately after sampling, leaf chlorophyll content was measured with a Soil Plant Analysis Development chlorophyll meter (Konica Minolta SPAD-502 meter) according to Uddling et al. (2007). Thereafter, leaves were stored in plastic bags on dry ice in the field and after returning to the lab in a freezer at -20°C until further analyses. Two leaves of each node were used per branch for bulk elemental analyses and one leaf was used for lipid analyses for each sampling date.

### 2.3 Bulk elemental and stable isotope (δ^13^C) analysis

The leaves were freeze-dried for the bulk elemental analyses using a Christ Alpha 1-2 lyophylisator and then manually crushed to fine powder using pestle and mortar under liquid nitrogen. The leaf samples were weighed into tin capsules and analysed for carbon (C), nitrogen (N), and stable carbon (δ^13^C) isotope composition using a Thermo Fisher Scientific Flash HT Elemental Analyser coupled to a Delta V Plus isotope ratio mass spectrometer *via* ConFlo IV and calibrated against several certified standards.

### 2.4 Lipid composition and characterisation

#### 2.4.1 Cuticular leaf wax preparation

Intact leaf samples were shaken for one minute in a solvent mixture of DCM : MeOH (99:1, v/v) and repeatedly washed with fresh solvent after being removed from the solvent reservoir (Wiesenberg and Gocke, 2017). Volume reduction was done via a Büchi Multivapor, followed by drying over anhydrous sodium sulfate (Na_2_SO_4_) and filtration using a glass-fiber filter. Afterwards, the solvent was evaporated until dryness to gravimetrically determine the concentration of cuticular waxes. The extract was then sequentially separated by solid phase extraction on activated silica gel (Wiesenberg and Gocke, 2017). Aliphatic and aromatic hydrocarbons were eluted with hexane and hexane:DCM (1:1, v/v), respectively, followed by low polar heterocompounds with DCM : MeOH (93:7, v/v). The volume was reduced via rotary evaporation (Wiesenberg and Gocke, 2017). Only aliphatic hydrocarbons were studied here in detail as they represent one of the most common components of leaf waxes (Ardenghi et al., 2017).

#### 2.4.2 Intracuticular leaf wax preparation

After extraction of the cuticular waxes, the air-dried extraction residues were crushed to fine powder with mortar and pestle under liquid nitrogen. A subsample of ~150mg of crushed leaf material was amended to ultrasonic extraction using 2ml of solvent mixture of DCM : MeOH (93:7, v/v) followed by centrifugation at 800*g* for 2 minutes, according to the procedure described by Wiesenberg and Gocke (2017). The supernatant was passed through a glass-fiber filter into a pre-weighed vial. This extraction was repeated 8 times. The solvent was evaporated in a fume hood until dryness and the intracuticular lipid concentration was determined gravimetrically. The lipid extract was re-dissolved in DCM and sequentially separated into neutral lipids, fatty acids (FA) and high molecular weight compounds after solid phase extraction with KOH-coated silica gel. Neutral lipids were eluted with DCM followed by FA with DCM:formic acid (99:1, v/v) and high molecular weight compounds with DCM : MeOH (1:1, v/v; Wiesenberg and Gocke, 2017). Out of these fractions, only FA are presented in this study as they are dominant in intracuticular leaf waxes and are a major precursor of *n*-alkanes which are predominant in the cuticular leaf waxes (Samuels et al., 2008). The cuticular and intracuticular lipid concentration was normalized by leaf area [µg.cm^-2^] due to the inverse proportionality of leaf dry weight and leaf area between sun-exposed and shaded leaves (**Supplementary Figure 1**; Prasad and Gülz, 1990; Huang et al., 2019).

#### 2.4.3 Fatty acid and alkane quantification

Deuterated standards (D_39_C_20_ or D_50_C_24_, respectively) were added to the individual FA and aliphatic hydrocarbon fractions for quantification before the respective analysis. Compound identification was performed on a gas chromatograph (GC; Agilent 6890) coupled to a mass selective detector (Agilent 5973N) equipped with a split/splitless injector. Injection was performed in splitless mode. Quantification was performed on a GC (Agilent 7890B) equipped with a multi-mode inlet and flame ionization detector (FID). The temperature program for the multi-mode inlet was ramped from 80°C (held for 0.5 min) to 400°C (held for 5 min) at 850°C/min and afterwards reduced to 250°C at 50°C/min. Both GC instruments were equipped with a J&W DB-5MS narrow-bore capillary column (50m x 0.2mm; 0.33µm film thickness) and a deactivated precolumn (1.5m). The oven temperature for *n*-alkane analyses increased from 70°C (held for 4 min) to 320°C (held for 20 min) at 5°C/min. The oven temperature program for FA started at 50°C (held for 4 min) and ramped to 150°C at 4°C/min and afterwards to 320°C (held for 40 min) at 3°C/min (Wiesenberg and Gocke, 2017).

#### 2.4.4 Compound-specific isotope analysis

The FA and *n*-alkane δ^13^C composition was determined using a Thermo Fisher Scientific Trace 1320 GC equipped with a PTV injector and FID detector connected *via* a GC-IsoLink II to a ConFlo IV and Delta V Plus isotope mass spectrometer (irMS). The temperature program of the PTV injector increased from 60°C (held for 0.5min) to 375°C (held for 2.5min) at 870°C/min and afterwards reduced to 250°C at 50°C/min. Injection was performed in splitless mode. The capillary column setup and the oven temperature program for both, FA and *n*-alkane fractions, was identical to the respective oven temperature programs used for quantification. All measurements were performed at least in triplicate and the difference between analytical measurements typically did not exceed 1‰ Vienna-Pee Dee Belemnite (V-PDB) for individual compounds. The CO_2_ reference gas was calibrated relative to V-PDB using A7 and B5 *n*-alkane and F8-3 fatty acid mixtures provided by A. Schimmelmann (Indiana University, Bloomington, Indiana, USA). Instrument performance was checked using an internal *n*-alkane standard mixture (C_20 -30_ Sigma Aldrich). The isotope (δ^13^C) ratio of FAs was corrected for the added methyl group during methylation (-42.0 ± 0.3‰).

### 2.5 Calculations

#### 2.5.1 Stable isotope composition and ^13^C-excess

The δ^13^C values are presented in per mil (‰) relative to the V-PDB standard:

1. *δ* ^13^ *C*=[((^13^ *C*/^12^ *C*_*leaf* *sample*_)/(^13^ *C*/^12^ *C*_*V*−*PDB*_))−1]*10^3^

Where ^13^C/^12^C_leaf sample_ is the ratio in the leaf sample and ^13^C/^12^C_V-PDB_ = 0.0112372

The ^13^C-excess is expressed as ^13^C atom% (Epron et al., 2012) and was calculated using the following equation:

2. ^13^ *C*−*excess*[*%*]=[(100/(^13^ *C*/^12^ *C*_*first* *leaf* *sample*_))*(^13^ *C*/^12^ *C*_*labelled* *leaf* *sample*_)]−100

#### 2.5.2 Molecular ratios

The average chain length (ACL) of *n*-alkanes and FA was calculated using the following equation (e.g. Wiesenberg and Gocke, 2017):

3. *ACL*= ∑^​^(*Z*_*n* _*n)/∑^​^(*Z*_*n*_) ,

where Z_n_ is the concentration of the respective compound and n is the number of carbon atoms; C_23-_C_31_ for *n*-alkanes (Griepentrog et al., 2016) and C_16_-C_32_ for FA.

### 2.6 Statistical analysis

Data represent mean values and standard errors (SE) of the same sampling date. C and N concentrations are calculated from eight replicates ± SE. The lipid concentration is given as mean value ± SE of four replicates (one control plus three labelled leaf samples) as there is no significant difference (*P*>0.05) in lipid concentration between control and labelled leaves for the individual sampling dates. The ^13^C-excess is provided as the weighted average of *n*-alkanes and FA ± SE of three replicates for all leaves. Only long-chain *n*-alkanes (C_25_, C_27,_ C_29_) were used to calculate the ^13^C-excess, as short-chain *n*-alkanes are lower concentrated. The ^13^C-excess of FA was calculated for chain-lengths between C_16_ and C_32_ as there was no significant difference (*P*>0.05) in the ^13^C-excess between short- and long-chain FA. Data analysis was performed with R studio software 4.0.4 (R Core Team, 2020). All data was tested for significant difference using one way analysis of variance (ANOVA; P<0.05).

## 3 Results

### 3.1 Leaf chemical properties

**Figure 2:**
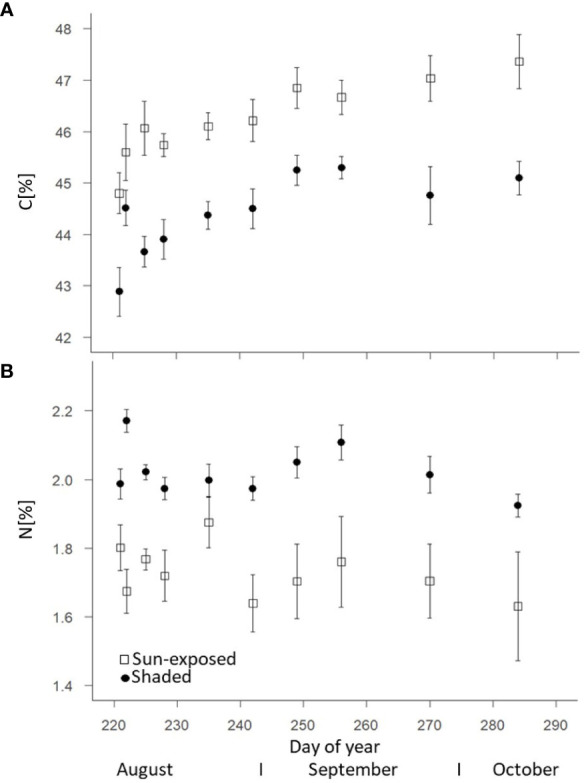
Change of the carbon **(A)** and nitrogen **(B)** concentrations of sun-exposed and shaded leaves during the late growing season (field replicates n=8 with 2 analytical replicates, each, error bars indicate SE).

Sun-exposed leaves showed a higher C concentration averaging 46.2 ± 1.3%, compared to shaded leaves that had an average C concentration of 44.4 ± 1.2% (+5%; F(2, 148) = 108.01, *P*<0.0001; **Figure 2A**). The C concentration increased in sun-exposed and shaded leaves from August (44.4 ± 1.1% and 42.8 ± 1.3%, respectively) to October (47.4 ± 1.4% and 45.1 ± 0.8%, respectively). Shaded leaves (2.02 ± 0.12%) showed a consistently higher N concentration compared to sun-exposed leaves (1.72 ± 0.26%) (+15%; F(2, 148) = 78.81, *P*<0.0001; **Figure 2B**). N concentration was highest in both, sun-exposed and shaded leaves at the beginning of August (1.87 ± 0.07% and 2.17 ± 0.03%, respectively). They declined until October (1.63 ± 0.15% and 1.92 ± 0.03%, respectively). Consequently, the C:N ratio increased from 27.7 ± 2.5 and 21.6 ± 0.5 in September to 32.1 ± 4.6 and 23.5 ± 0.3 in October in sun-exposed (+15%) and shaded (+10%) leaves (**Supplementary Figure 2**). SPAD-values indicative for the chlorophyll content of sun-exposed (31.7 ± 4.2) and shaded (31.8 ± 1.8) leaves did not differ between these exposures (F(2, 149) = 0.12, *P*=0.73) over the late growing season (**Supplementary Figure 3**). The SPAD-values decreased in both, sun-exposed (r = -0.504; R^2^ = 0.254) and shaded leaves (r = -0.594; R^2^ = 0.353) from August to October. Water concentration was consistently higher (F(2, 144) = 83.43, *P*<0.0001) in shaded (57.7 ± 3.4%) than in sun-exposed leaves (51.7 ± 4.7%). They remained almost constant during the late growing season (**Supplementary Figure 4**).

### 3.2 Lipid composition

**Figure 3:**
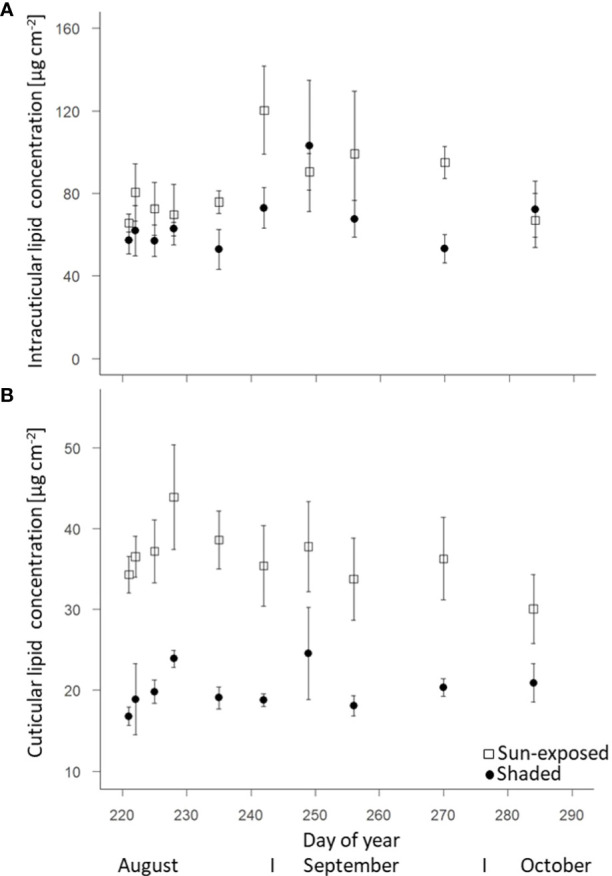
Change of intracuticular **(A)** and cuticular **(B)** lipid concentrations of sun-exposed and shaded leaves during late growing season (field replicates n = 4, error bars indicate SE).

The total intracuticular lipid concentration was higher (+20%; F(2, 69) = 7.60, *P*<0.01) in sun-exposed (83.6 ± 4.9 µg.cm^-2^) than in shaded leaves (66.1 ± 4.3 µg.cm^-2^; **Figure 3A**). Sun-exposed leaves (36.4 ± 1.4 µg.cm^-2^) had a higher cuticular lipid concentration (+45%; F(2, 68) = 109.29, *P*<0.0001) compared to shaded leaves (19.9 ± 0.7 µg.cm^-2^; **Figure 3B**). The cuticular lipid concentration tended to decrease in sun-exposed leaves (-12%; r = -0.156; R^2^ = 0.024), while it tended to increase in shaded leaves (+20%, r = 0.132; R^2^ = 0.017) during the late growing season.

**Figure 4:**
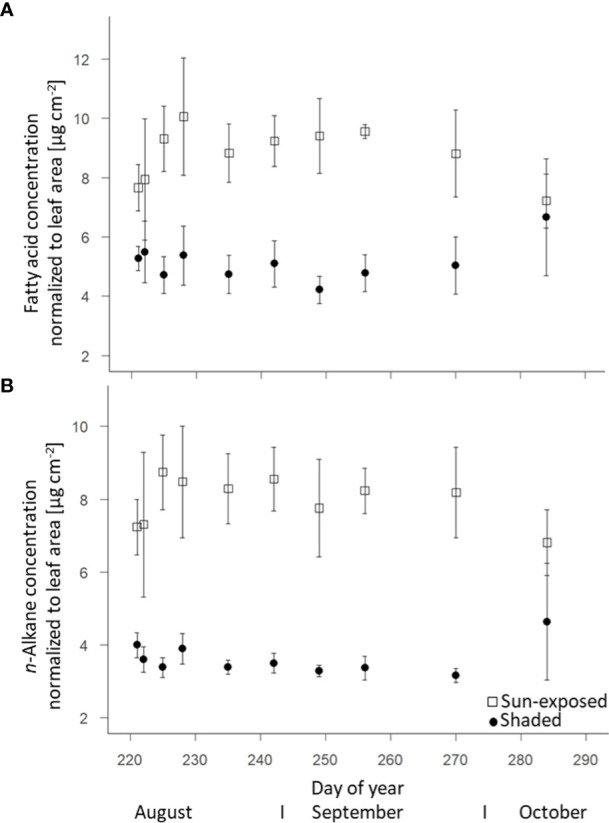
Change of fatty acids (intracuticular waxes) **(A)** and *n*-alkane (cuticular waxes) **(B)** concentrations of sun-exposed and shaded leaves during the late growing season (field replicates n=4, error bar indicate SE).

Sun-exposed leaves (8.7 ± 0.4 µg.cm^-2^) were characterized by a higher intracuticular FA concentration (+45%; F(2, 63) = 57.55, *P*<0.0001) than shaded leaves (5.2 ± 0.5 µg.cm^-2^). The FA concentration in sun-exposed leaves was highest (10.1 ± 0.7 µg.cm^-2^) in early August (DOY228). Thereafter, the FA concentration decreased (-5%; 7.2 ± 0.9 µg.cm^-2^) towards the end of the growing season (**Figure 4A**). In contrast, the FA concentration in shaded leaves remained almost constant over the late growing season. In line with the lipid concentration, sun-exposed leaves (7.9 ± 0.3 µg.cm^-2^) contained a constantly higher *n*-alkane concentration in the cuticula (+52%, F(2, 68) = 123.50, *P*<0.0001) than shaded leaves (3.8 ± 0.3 µg.cm^-2^; **Figure 4B**). No temporal trend was observed.

FA showed a strong even-over-odd dominance with a chain length ranging from C_16:0_ to C_32:0,_ with C_16:0_, C_18:3_, C_20:0_ and C_28:0_ being the most abundant. A similar concentration (t(74) = -0.32 *P*=0.75) of C_16:0_ was found in both sun-exposed (33.4 ± 1.9%) and shaded (32.6 ± 1.8%) leaves (**Supplementary Figure 5A**). Shaded leaves had a higher (t(59) = 5.17, *P*<0.001) concentration of C_18:3_ (10.1 ± 1.1%) compared to sun-exposed leaves (6.1 ± 0.7%). Sun-exposed leaves had a higher (t(73) = -3.95, *P*<0.005) concentration of C_20:0_ (15.5 ± 0.7%) and C_28:0_ (16.6 ± 1.1%) compared to shaded leaves (9.9 ± 0.4% and 12.3 ± 1.1%, respectively). The average chain length of FA was consistently higher in sun-exposed than in shaded leaves (F(2, 64) = 115.54, *P*<0.0001) (**Supplementary Figure 6A**).

In all investigated leaf samples, *n*-alkanes had a strong odd-over-even dominance with alkanes ranging from C_23_ to C_31_ (**Supplementary Figure 5B**). *n*-C_27_ was the most abundant (ca. 90%) alkane in all leaves. Sun-exposed leaves (3.5 ± 0.1%) exhibited a lower concentration of *n*-C_29_ (t(76) = -4.06, P< 0.001) compared to shaded leaves (5.6 ± 0.1%). At the same time, the concentration of *n*-C_25_ was higher (t(76) = -11.09, P< 0.001) in sun-exposed (3.1 ± 0.0%) than in shaded (2.5 ± 0.0%) leaves. This resulted in a lower average chain length (-1%; F(2, 68) = 177.00, *P*<0.0001) of sun-exposed leaves (26.9 ± 0.1) than of shaded leaves (27.1 ± 0.1; **Supplementary Figure 6B**).

### 3.3 Stable carbon (δ^13^C) isotope composition

**Figure 5:**
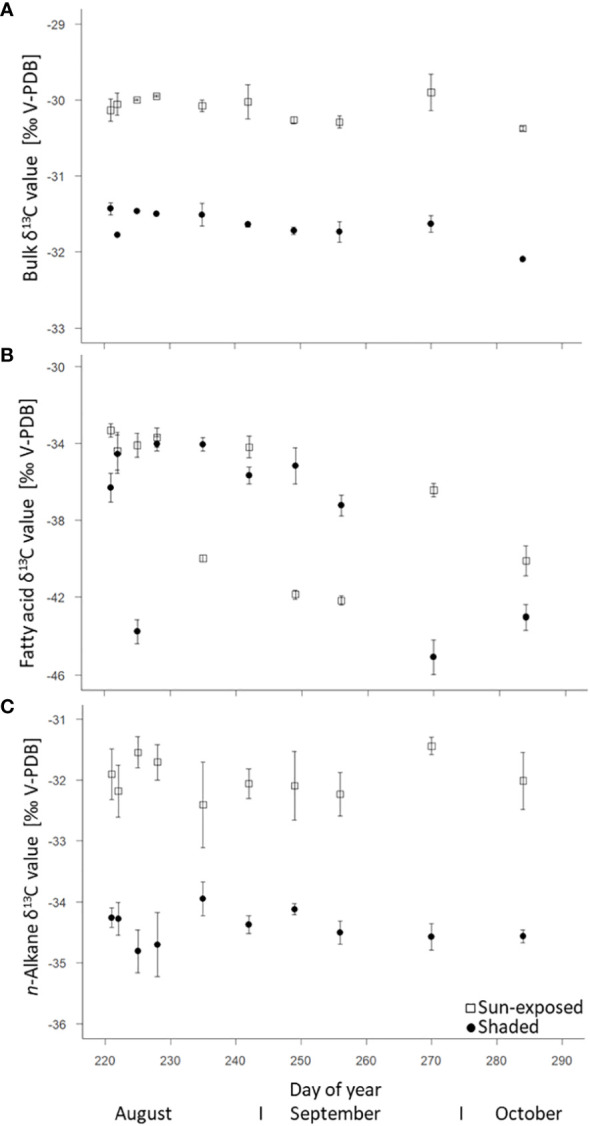
Natural δ^13^C abundance (control spot) of bulk tissue **(A)**, fatty acids (intracuticular waxes) **(B)**, and *n*-alkanes (cuticular waxes) **(C)** of sun-exposed and shaded leaves (analytical replicates n=3, error bars indicate SE).

Sun-exposed leaves always revealed higher bulk δ^13^C values (+1.5‰; F(2, 29) = 1051.60, *P*<0.0001) compared to shaded leaves with average values of -30.1 ± 0.2‰ and -31.6 ± 0.2‰, respectively (**Figure 5A**). In general, there was little variation ( ± 1‰) in the bulk δ^13^C values over the late growing season. There was no significant difference (F(2, 9) = 0.23, *P*=0.64) in the δ^13^C values of FA between sun-exposed (-37.0 ± 0.5‰) and shaded leaves (-37.8 ± 0.6‰). More negative δ^13^C values of FA of all leaves were observed towards the end of the growing season. FA showed a higher intra-seasonal variability in the δ^13^C values (SD=1.20) and a depletion of 3-6‰ with respect to bulk δ^13^C values (SD = 0.13; **Figure 5B**). This was also observed for *n*-alkane δ^13^C values (**Figure 5C**), but to a lesser extent. The δ^13^C values of *n*-alkanes showed a depletion of 1-3‰ respective to bulk δ^13^C values. An enrichment in ^13^C by +2.6‰ (F(2, 45) = 247.39, *P*<0.0001) was observed for *n*-alkanes of sun-exposed leaves compared to shaded leaves.

### 3.4 ^13^C-excess of sun-exposed and shaded leaves

**Figure 6:**
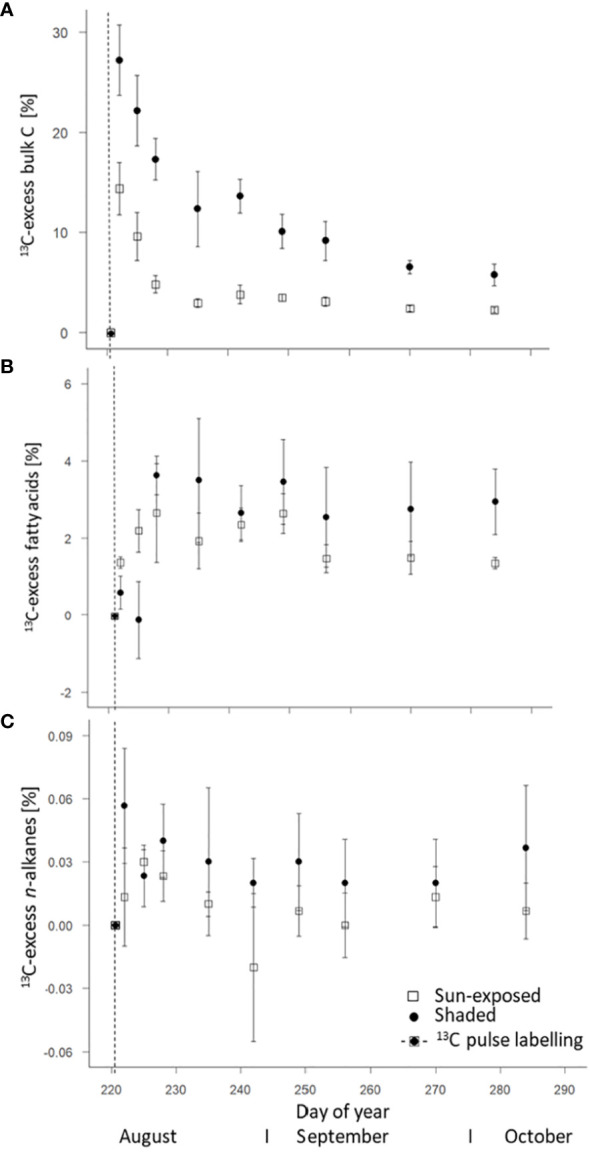
Weighted average of ^13^C-excess of bulk C **(A)**, fatty acids (intracuticular waxes) **(B)**, and *n*-alkanes (intracuticular waxes) **(C)** of sun-exposed and shaded leaves after ^13^CO_2_ pulse.

The ^13^C-excess of both, sun-exposed and shaded leaves was calculated based on the ^13^C/^12^C ratio from the first leaf sample at time t=0 of the respective branch. The maximum ^13^C-excess was observed in bulk leaves one day (DOY 222) after labelling with a ^13^C-excess of 14 ± 3% in sun-exposed and 27 ± 3% in shaded leaves (**Figure 6A**). Overall, the ^13^C-excess was lower (F(2, 108) = 78.24, *P*<0.0001) in sun-exposed than in shaded leaves. A faster exponential decrease occurred in sun-exposed (r = -0.773; R^2^ = 0.597) than in shaded leaves (r = -0.397; R^2^ = 0.157) towards the late end of the growing season.

FA of sun-exposed and shaded leaves reached an average maximum in ^13^C one week after labelling (DOY 228). ^13^C-excess was lower (F(2, 45) = 0.07, P=0.78) in sun-exposed (2.65 ± 1.27%) than in shaded leaves (3.63 ± 0.49%; **Figure 6B**). Normalized to the ^13^C-excess in the respective bulk leaf, the sun-exposed leaves invest more (18%) of the assimilated C into FA and secondary metabolites than shaded leaves (13%). The enrichment in ^13^C in sun-exposed leaves is visible immediately after labelling whereas the ^13^C enrichment in shaded leaves occurred more slowly. Moreover, the ^13^C enrichment in shaded leaves remained almost constant until late growing season. On the contrary, the ^13^C enrichment in sun-exposed leaves tended to decrease in the late growing season. High variability was observed among individual branches (**Supplementary Figure 7**).

**Figure 7:**
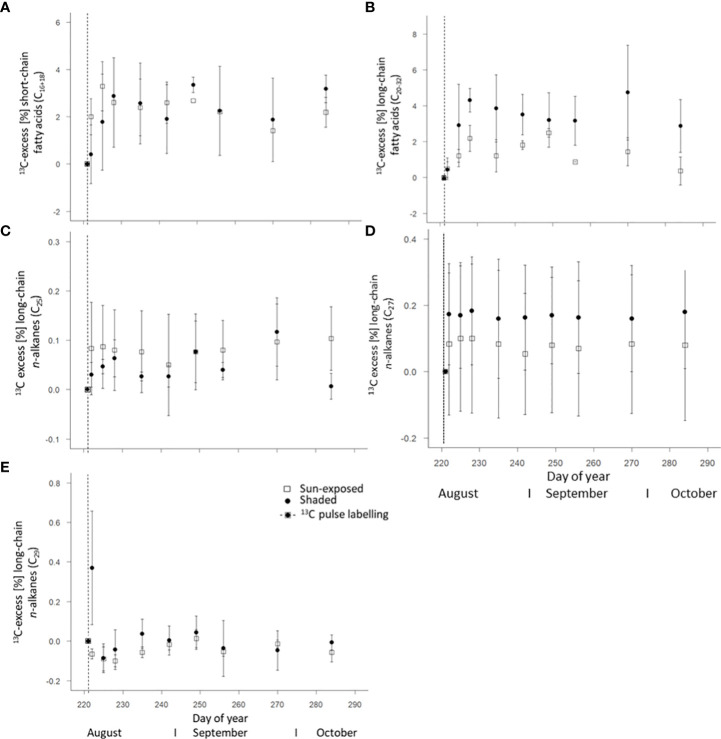
Weighted average of ^13^C-excess of short-chain fatty acids C_16-18_ **(A)**, long-chain fatty acids C_20:0-32:0_ **(B)** of intracuticular waxes as well as *n*-alkanes C_25_ **(C)**, C_27_ **(D)**, and C_29_ **(E)** of the cuticular waxes of sun-exposed and shaded leaves (analytical replicates n=3, error bars indicate SE). The vertical line in the graphs indicates the application date of the isotope label.

Short-chain FA (C_16:0 and 18:0_) showed a maximum in ^13^C four days (DOY225) and one week (DOY228) after labelling in sun-exposed and shaded leaves with a ^13^C-excess of 3.29 ± 1.04% and 2.88 ± 0.37%, respectively (**Figure 7A**). Within an individual branch, however, the ^13^C-excess of short-chain FA in shaded branches remained constant after reaching a maximum of ^13^C-excess. The ^13^C-excess of short-chain FA in sun-exposed branches, however, showed a decay before stabilization. There was no significant difference (F(2, 45) = 0.09, *P*=0.76) in the ^13^C-excess of short-chain FA between sun-exposed and shaded leaves throughout the entire late growing season. This is caused by a high variability in the ^13^C-excess of short-chain FA among all branches (**Supplementary Figures 7C, D**). Long-chain FA (C_20:0 to 32:0_) of all branches showed a first maximum in ^13^C one week after labelling (DOY228) with a ^13^C-excess of 2.17 ± 0.72% in sun-exposed and of 4.31 ± 0.65% in shaded leaves, respectively (**Figure 7B**). Overall, long-chain FA of shaded leaves had a larger ^13^C-excess (F(2, 45) = 10.08, *P*<0.01) than those of sun-exposed leaves. There was a high variability in the ^13^C-excess of long-chain FA among shaded branches. However, there was no significant difference (F(3, 15) = 1.65, *P*=0.23) in the ^13^C-excess of long-chain FA among sun-exposed branches (**Supplementary Figures 7E, F**).

The weighted average of ^13^C-excess of *n*-alkanes in all leaves showed a low enrichment in ^13^C in comparison to the *n*-alkane δ^13^C values of the unlabelled leaves with a lower ^13^C-excess (F(2, 49) = 4.04, *P*<0.05) in sun-exposed (0.01 ± 0.01%) than in shaded leaves (0.03 ± 0.01%; **Figure 6C**). There was no significant difference in the ^13^C-excess of *n*-C_25_ (F(2, 49) = 1.60, *P*=0.21) and *n*-C_29_ (F(2, 48) = 2.58, *P*=0.11) between sun-exposed and shaded leaves (**Figures 7C, D**). Shaded leaves, however, had a larger ^13^C-excess for *n*-C_27_ (F(2, 49) = 4.84, *P*< 0.05) compared to sun-exposed leaves (**Figure 7E**). Overall, there was a high variability among individual branches in the ^13^C-excess for long-chained *n*-alkanes (*n*-C_25_ to *n*-C_27_) in shaded leaves (F(3, 18) = 17.06, *P*<0.0001). There was no significant difference in the ^13^C-excess among sun-exposed branches (F(3, 18) = 2.53, *P*=0.11; **Supplementary Figures 8A–F**).

Over the entire late growing season, the ^13^C-excess was largest for bulk leaves (10-30%; one day after labelling) followed by FA (1-4%; one week after labelling) and *n*-alkanes (0.01-0.04%; within the first week after labelling if any). In general, over the entire late growing season, ^13^C-excess of *n*-alkanes and FA followed a similar trend.

## 4 Discussion

### 4.1 Differences between sun-exposed and shaded leaves

Compared to sun-exposed leaves, shaded leaves are characterized by larger leaf area, thinner leaves (Van Wittenberghe et al., 2012), and higher foliar chlorophyll as well as nitrogen concentration. This is necessary to successfully intercept diffuse radiation and maintain photosynthesis despite light limitation (Bachofen et al., 2020). Consequently, this explains that shaded leaves showed consistently higher N concentration (+15%; F(2, 148) = 78.81 *P*<0.0001; **Figure 2B**) over the entire late growing season in our study. The higher C concentration (+5%; F(2, 148) = 108.01, *P*<0.0001) in sun-exposed (46.2 ± 1.3%) over shaded leaves (44.4 ± 1.2%) can be attributed to the larger concentration of carbon-rich compounds such as soluble sugars (Castrillo et al., 2005). Those concentrations often coincide with photosynthesis rates. The observed bulk δ^13^C values (**Figure 5A**) of leaves varying between -30 and -32‰ are identical to those reported by Schleser (1990) for beech leaves. This observed ^13^C depletion (-1.5‰; F(2, 29) = 1051.60, *P*<0.0001) of shaded compared to sun-exposed leaves can be explained by stomatal closure and a lower mesophyll conductance in sun-exposed leaves to minimize water loss. This typically results in less ^13^C discrimination (Cano et al., 2013). As a result of the sun exposure and higher water loss, sun-exposed leaves were characterized by a consistently lower (F(2, 144) = 83.43, *P*<0.0001) water concentration compared to shaded leaves (**Supplementary Figure 3**; Pilegaard et al., 2003). Therefore, within an individual tree, leaf functional traits are diverse (Forey et al., 2016) and partly driven by sun exposure.

As a result of sun exposure, differences in abundance and composition of leaf lipids can also be expected within the canopy (Bush and McInerney, 2013). Sun-exposed leaves had an overall higher concentration of cuticular lipids (+45%; F(2, 68) =109.29, *P*<0.0001) compared to shaded leaves (**Figure 3B**). Specifically, the higher *n*-alkane concentration per leaf area (+52%, F(2, 68) = 123.50, *P*<0.0001; **Figure 4B**) in sun-exposed compared to shaded leaves suggest a relation between *n*-alkane biosynthesis and exposure to solar radiation and low water content. This higher *n*-alkane concentration in sun-exposed compared to shaded leaves confirms our first hypothesis of a higher *n*-alkane concentration in sun-exposed over shaded leaves. Furthermore, our findings are consistent with results on the seasonal dynamic of the cuticular wax composition (Prasad and Gülz, 1990). In contrast, the results of lower (-1%; F(2, 68) = 177, *P*<0.0001) ACL values in sun-exposed versus shaded leaves contradict the second part of our first hypothesis (i) of greater ACL values in sun-exposed than shaded leaves (**Supplementary Figures 5A, B**). This result further contradicts several studies that reported a positive correlation between *n*-alkane chain lengths and increased temperature (Bush and McInerney, 2013; Tipple and Pagani, 2013). An explanation for the lower *n*-alkane ACL values in sun-exposed leaves within this study might be the faster *n*-alkane biosynthesis of *n*-C_25_ vs. *n*-C_27_-alkanes caused by environmental stress that results in a reduced chain-elongation (Post-Beittenmiller, 1996). This faster lipid metabolism could lead to the formation of *n*-alkanes with a lower chain-length as a result of incomplete enzymatic chain elongation (Leonard et al., 2004) as observed in grass species (Srivastava and Wiesenberg, 2018). Consequently, this argues for plant species specific responses of biosynthetic lipid synthesis depending on environmental stress.

### 4.2 Intra-seasonal trends of leaf properties over the late growing season

The intracuticular (**Figure 3A**) and cuticular lipid (**Figure 3B**) concentrations as well as the FA (**Figure 4A**) and *n*-alkane (**Figure 4B**) concentrations of sun-exposed leaves decreased from September onwards. At a first glance, this confirmed previous observations for *n*-alkanes (Sachse et al., 2009). This can indicate a progressive wax degradation (Nguyen-Tu et al., 2007) that is likely coupled to an overall reduction in wax biosynthesis as reported by Sachse et al. (2015) for oak trees. It corroborated the on-going leaf senescence evidenced by a decline in SPAD-derived chlorophyll content (**Supplementary Figure 2**), and N concentration (**Figure 2B**). This is related to a decrease in photosynthetic activity, as well as a decline in water concentration (**Supplementary Figure 3**) throughout the late growing season. This trend was more pronounced in sun-exposed compared with shaded leaves whose senescence started later. In contrast to sun-exposed leaves, the intracuticular and cuticular wax lipid concentration of shaded leaves increased between September and October, indicating an ongoing lipid biosynthesis. This might have been prompted by the leaf fall starting from the outermost parts of the crown, resulting in the exposure of shaded leaves to sunlight conditions during this late growing season. As reported by Giese (1975), leaves grown previously under light deficiency exhibited the same amount of cuticular wax lipids as light grown leaves after being exposed to light conditions for 24 hours. Consequently, there is a high variability in *n*-alkane and FA concentrations between September and October when previously shaded leaves became exposed to sunlight. Therefore, this partially contradicts our second hypothesis (ii) of a higher variability in *n*-alkane and FA concentrations over the late growing season in sun-exposed compared to shaded leaves.

The observed ^13^C-depletion of FA (-5‰) and *n*-alkanes (-3‰, **Figures 5A–C**) compared with bulk C are in agreement with results of other studies that reported a ^13^C depletion in lipids between 2 to 9‰ relative to bulk tissue (Ballentine et al., 1998; Hobbie and Werner, 2004). This depletion can be explained by isotope fractionation during the oxidation of pyruvate to coenzyme-A in lipid biosynthesis (DeNiro and Epstein, 1977; Collister et al., 1994). The decline in *n*-alkane δ ^13^C in all leaves towards the end of the growing season (**Figure 5B**) suggested an ongoing replacement of leaf wax lipids (Lockheart et al., 1997) that can be removed during abrasion (Conte et al., 2003) or other processes. Generally, we observed a significant difference (F(2, 45) = 247.39, *P*<0.0001) in *n*-alkane δ^13^C values between sun-exposed and shaded leaves, as sun-exposed leaves revealed higher variability of δ^13^C values over the entire late growing season. This can be related to changes in the photosynthetic assimilation rate in response to sun exposure (Lockheart et al., 1997). Further, this partially confirms our second hypothesis (ii) of a higher variability in *n*-alkane isotope composition in sun-exposed compared to shaded leaves. Surprisingly, and in contrast to bulk and *n*-alkane δ^13^C values, our results showed no significant difference (F(2, 9) = 0.23, *P*=0.64) between the δ^13^C values of FA of sun-exposed and shaded leaves (**Figure 5C**). Thus, the δ^13^C values of FA behave differently compared to bulk and *n*-alkanes δ^13^C values in all leaves, which was contrary to our expectation. Since bulk tissues as well as lipids of sun-exposed leaves typically have higher δ^13^C values than their shaded counterparts, the same can be expected for δ^13^C values of FA. As this could not be observed, this might suggest a partial decoupling of FA biosynthesis from alkane biosynthesis, as FA are precursors of a wide range of secondary metabolites (Post-Beittenmiller, 1996). However, this hypothesis could not be elucidated in the current study and therefore requires further investigation.

### 4.3 Ongoing lipid biosynthesis

The ^13^C labelling experiment followed by the subsequent assessment of the ^13^C-excess in bulk C, individual FA and *n*-alkanes provided new insights into the C and lipid renewal taking place in leaves of a mature deciduous trees. The dependency of wax lipid renewal on sun exposure could be determined by the chosen approach.

The change of the natural δ^13^C abundance of ~10‰ in the FA (**Figure 5B**) observed during the late growing season indicate a high within-individual heterogeneity and a potential rapid leaf lipid formation and replacement within the investigated beech tree. This is in good agreement with the study of Sachse et al. (2009), who found a continuous leaf wax biosynthesis in temperate forest species (*Acer pseudoplantanus* and *Fagus sylvatica*) determined by compound-specific δ^2^H analysis of *n*-alkanes. The seasonal variability in the ACL values of FA (**Supplementary Figure 5A**) within the late growing season, specifically in shaded leaves, further supports a *de-novo* biosynthesis of wax lipids. Such observations were only partially made before for wax crystals (Neinhuis et al., 2001) and *n*-alkanes (Tipple et al., 2013). The rapid increase of the ^13^C-excess of short-chain and long-chain FA (**Figures 7A, B**) within a few days after the short-term isotopic pulse of 2-4% further indicates substantial *de-novo* lipid biosynthesis within the late growing season. Furthermore, the ratio of the ^13^C-excess of the bulk leaves to the ^13^C-excess of the FA of the respective leaf suggests a larger investment of the assimilated C into FA and secondary metabolites in sun-exposed than in shaded leaves. One potential substance class that benefitted from this ongoing FA biosynthesis was potentially the cutin polymer (Kolattukudy and Walton, 1973) or plant pigments that also showed considerable changes in their composition during the same observation period for the whole tree (Petibon and Wiesenberg, 2022). As ^13^C excess of FA exhibited a subsequent decrease until the end of the observation period and some variability of individual sampling dates, this supports our third hypothesis (iii) of a *de-novo* biosynthesis of leaf wax lipids in a mature beech tree of ~200 years even within the late phase of the growing season.

In contrast to FA, the very low ^13^C-excess of *n*-alkanes suggests a minor or almost no incorporation of ^13^C from the labelling experiment into leaf *n*-alkanes during the late growing season. This difference can be explained by the fact that FA are intermediates and *n*-alkanes are end products within biosynthesis (Kunst and Samuels, 2003), which is why for the latter smaller changes can be expected (Wiesenberg et al., 2010). Overall, we observed minor changes in *n*-alkane concentration (**Figure 4B**), average chain length of *n*-alkanes (**Supplementary Figure 5B**), and compound-specific δ^13^C values of *n*-alkanes, which confirms ongoing biosynthesis of alkanes during the late growing season. This is in line with observations made by Sachse et al. (2009) for δ^2^H values of tree *n*-alkanes. Some previous studies on leaf *n*-alkane biosynthesis on *Populus trichocarpa* and *Populus angustifolia* under controlled conditions highlighted the decisive role of leaf *n*-alkane biosynthesis in early leaf growth stage (Kahmen et al., 2011; Tipple and Pagani, 2013). Our results argue that alkane biosynthesis continues even during late phases of the growing season, however, to a lesser extent than FA biosynthesis. Aside from the compositional changes, the variability in the ^13^C-excess in long-chained (*n*-C_25_ and *n*-C_29_) *n*-alkanes (**Figures 7C and D**) also support these findings. Due to the observed small changes of *n*-alkane composition and ^13^C-excess close to 0 and thus within the statistical error, it remained difficult to calculate wax renewal rates in our experiment. Therefore, follow-up experiments might gain a higher ^13^C-enrichment by multiple ^13^C-pulses or try continuous *in-situ* isotope labelling over several days to better trace the isotope label. However, this could not be achieved in our study, which was one of the first of its kind, where a ^13^CO_2_ pulse was applied on branches of mature trees and the isotope label was tried to follow at a molecular level, thereafter.

## 5 Conclusion

The leaf lipid composition in a mature (~200-year-old) beech tree was traced, aided by a ^13^C-pulse-chase labelling experiment in combination with compound-specific isotope analyses to investigate the late-season dynamic (August until October) of carbon uptake and renewal of leaf wax lipids in relation to sun exposure. In sun-exposed leaves, FA and *n*-alkane concentrations per leaf area were higher than in shaded leaves offering a better protection against UV radiation, heat and water loss. We found a continuous renewal of fatty acids within leaves and to a lesser extent *n*-alkanes reflected by compositional changes and ^13^C-excess. Consequently, the continuous renewal of wax lipids supports the hypothesis of an ongoing maintenance of the cuticular functionality and the ability of mature tree leaves to respond to changing environmental conditions even during the late growing season. Despite of compositional changes, the overall wax content tended to remain constant over time. This argues for a continuous replacement of fatty acids within the leaf and *n*-alkanes at the leaf surface at the expense of a simultaneous FA conversion and loss of alkanes at the leaf surface. This further suggests a continuous release of wax *n*-alkanes into the environment, contributing to aerosols and deposition of *n*-alkanes in soils and sediments throughout the whole growing season. As FA were replaced faster than alkanes, this implies that a large portion of long-chain FA are further used for the formation of other biosynthates, such as pigments or cutin polymers. The current study could successfully prove the ongoing biosynthesis of plant waxes of mature deciduous trees. However, for more systematic and more holistic study of plant physiological processes, larger branches and with a higher number of samples and sample amounts should be considered to study for more holistic study of the various plant constituents and processes.

## Data availability statement

The original contributions presented in the study are included in the article/**Supplementary Material**. Further inquiries can be directed to the corresponding author.

## Author contributions

Conceptualization: FP, GW; Project administration, Funding acquisition and Resources: GW; Supervision: FP, GW; Investigation & Data curation: TS; Methodology: FP, GW, TS; Formal Analysis & Visualization: TS; Writing – original draft: TS; Writing – review & editing: FP, GW, TS. All authors contributed to the article and approved the submitted version.
