# Supplementary material for: Late-season biosynthesis of leaf fatty acids and n-alkanes of a mature beech (Fagus sylvatica) tree traced via 13CO2 pulse-chase labelling and compound-specific isotope analysis
Source: Front Plant Sci. 2023 Jan 6;13:1029026. doi: 10.3389/fpls.2022.1029026 (PMC9853289; doi:10.3389/fpls.2022.1029026)

Supplementary Material

**Supplementary Figure 1** Leaf dry weight [mg] and leaf area [cm^2^] of sun-exposed and shaded leaves (n = 160).


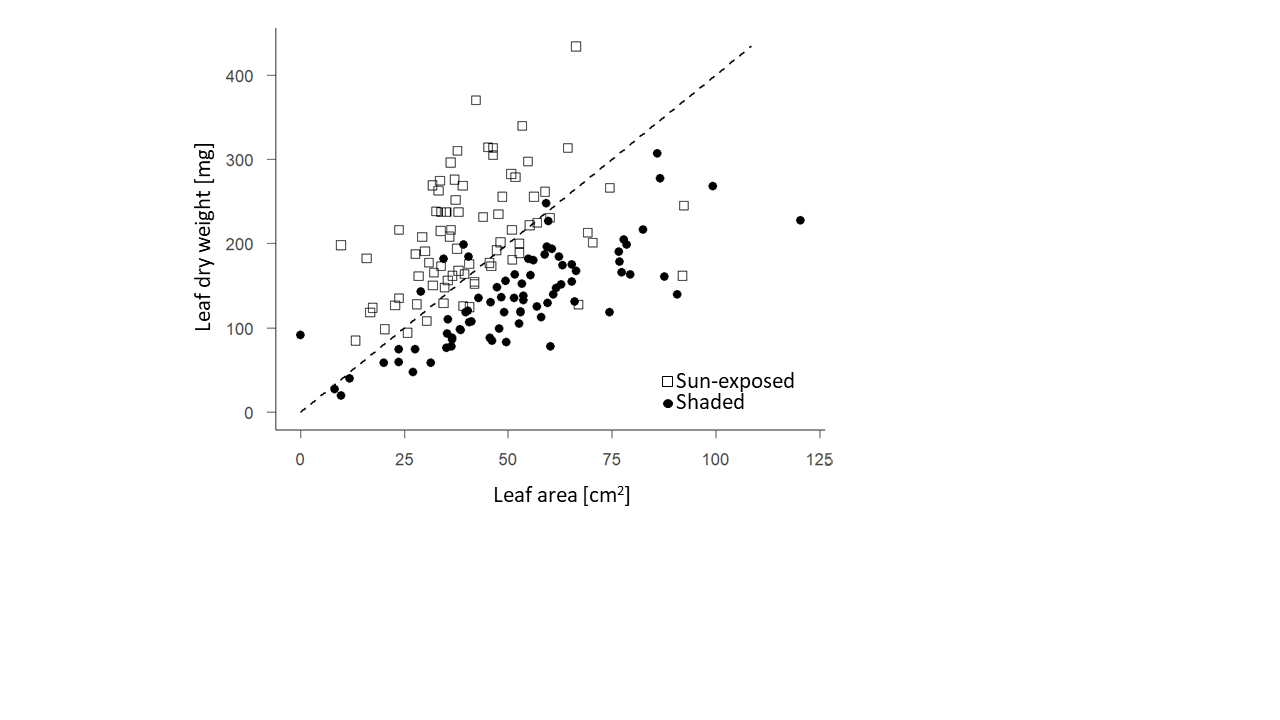

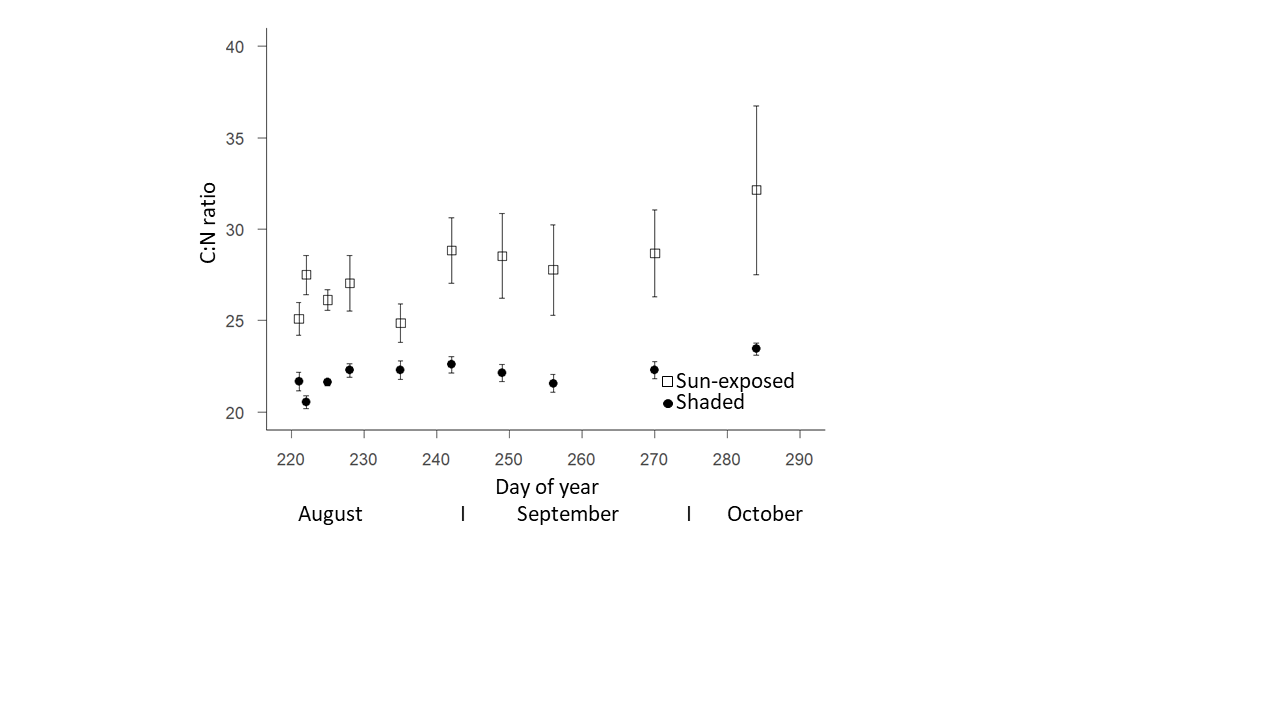


**Supplementary Figure 2** C:N ratio of sun-exposed and shaded leaves during the late growing season (field replicates n=8 with 2 analytical replicates, each, error bars indicate SE).

**Supplementary Figure 3** Change of SPAD values of sun-exposed and shaded leaves during the late growing season (field replicates n=8, error bars indicate SE).


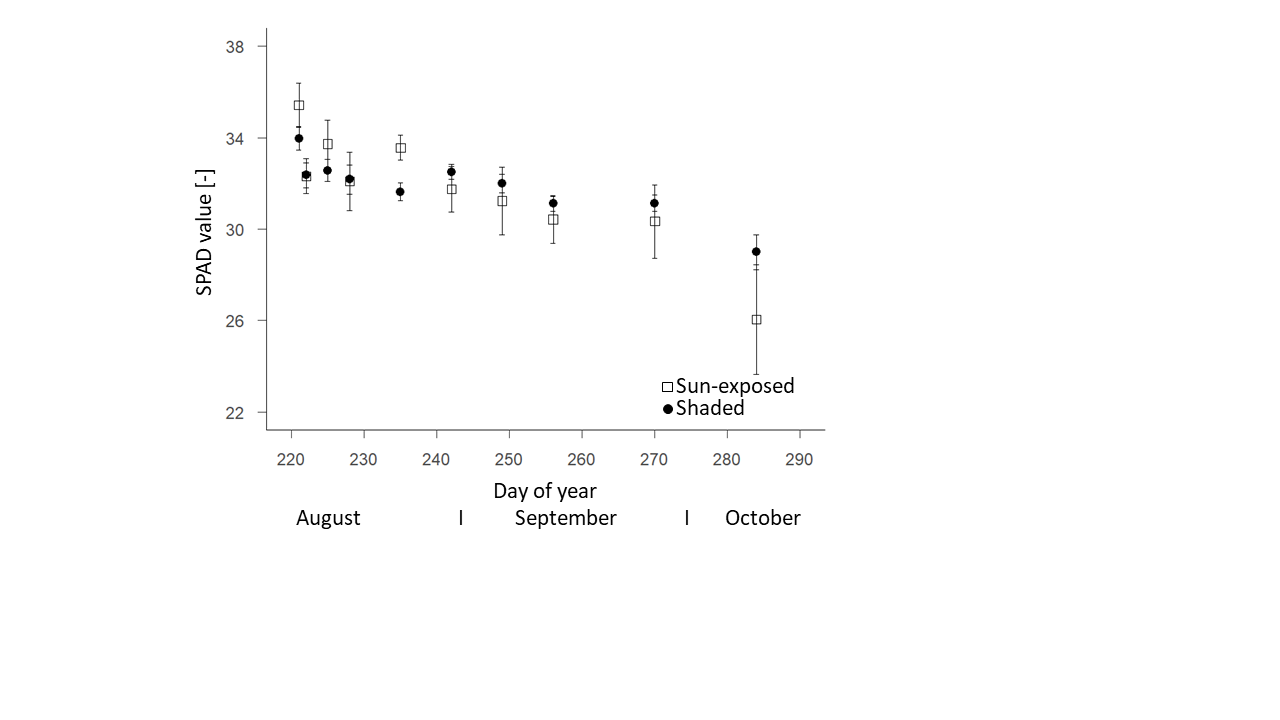


**Supplementary Figure 4** Change of water concentration of sun-exposed and shaded leaves during the late growing season (field replicates n=8, error bars indicate SE).


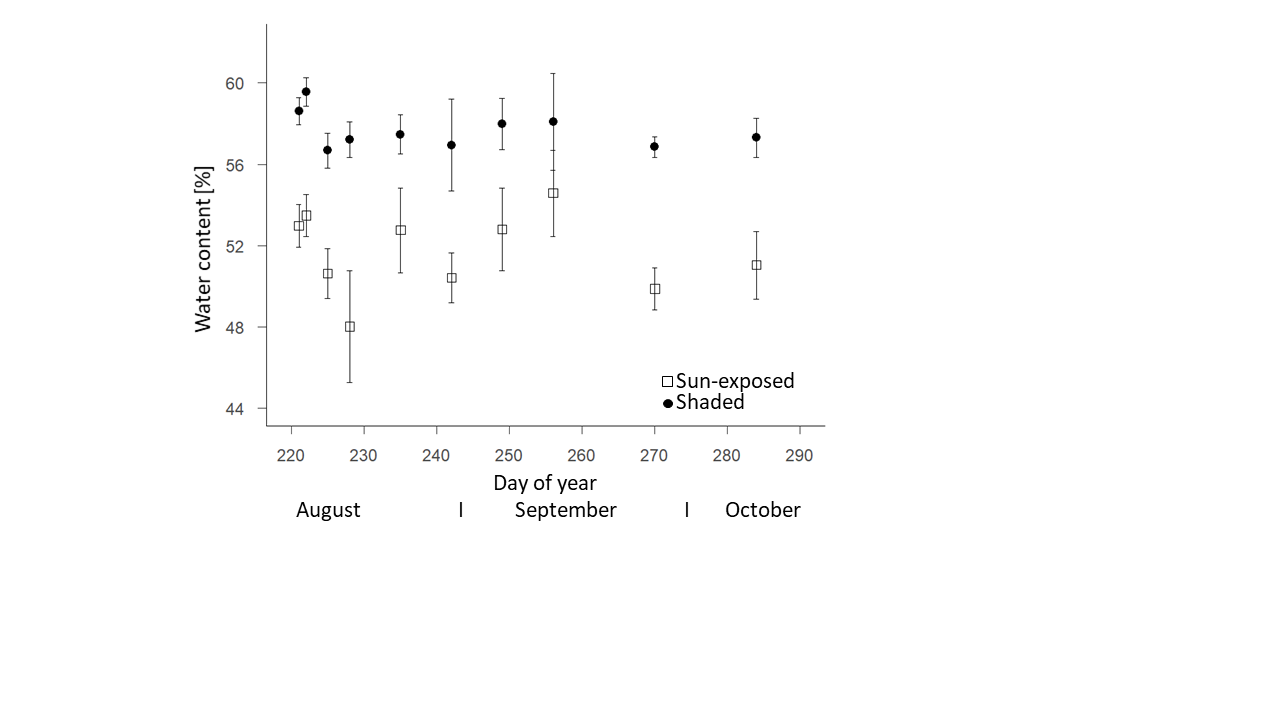

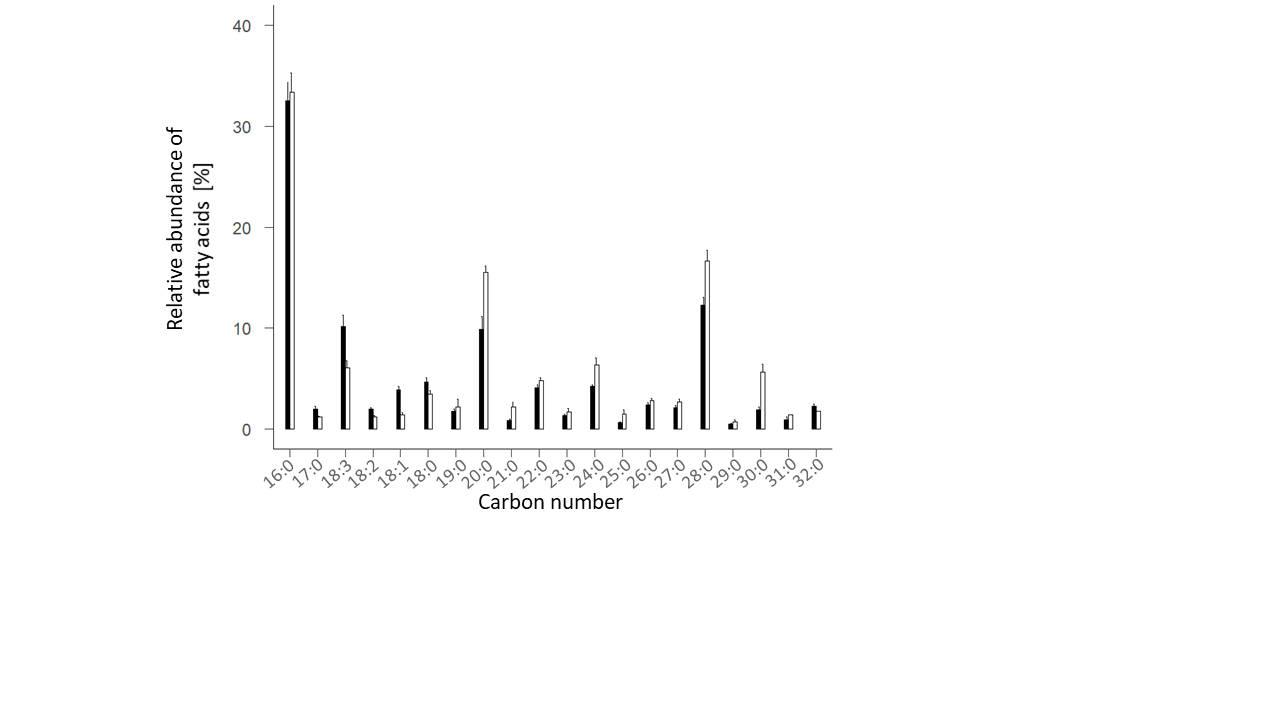

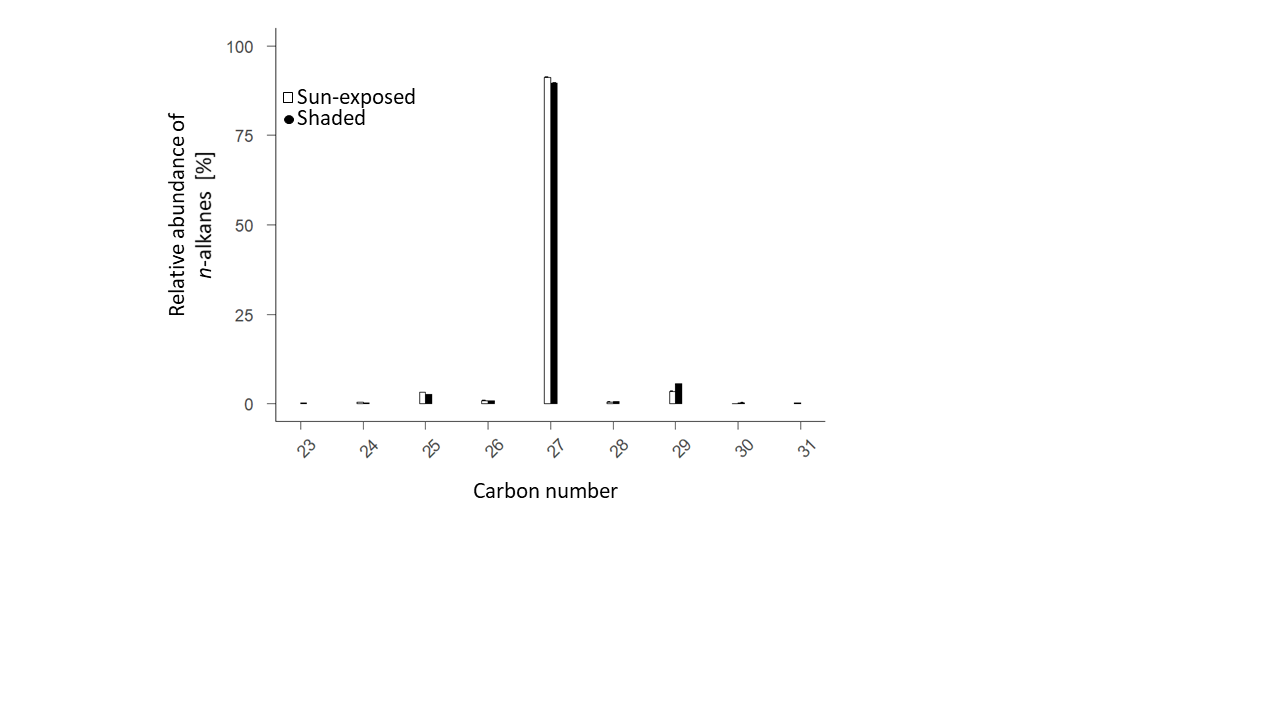


**Supplementary Figure 5** Relative abundance of fatty acids (a) and *n*-alkanes (b) of sun-exposed and shaded leaves

a)

b)


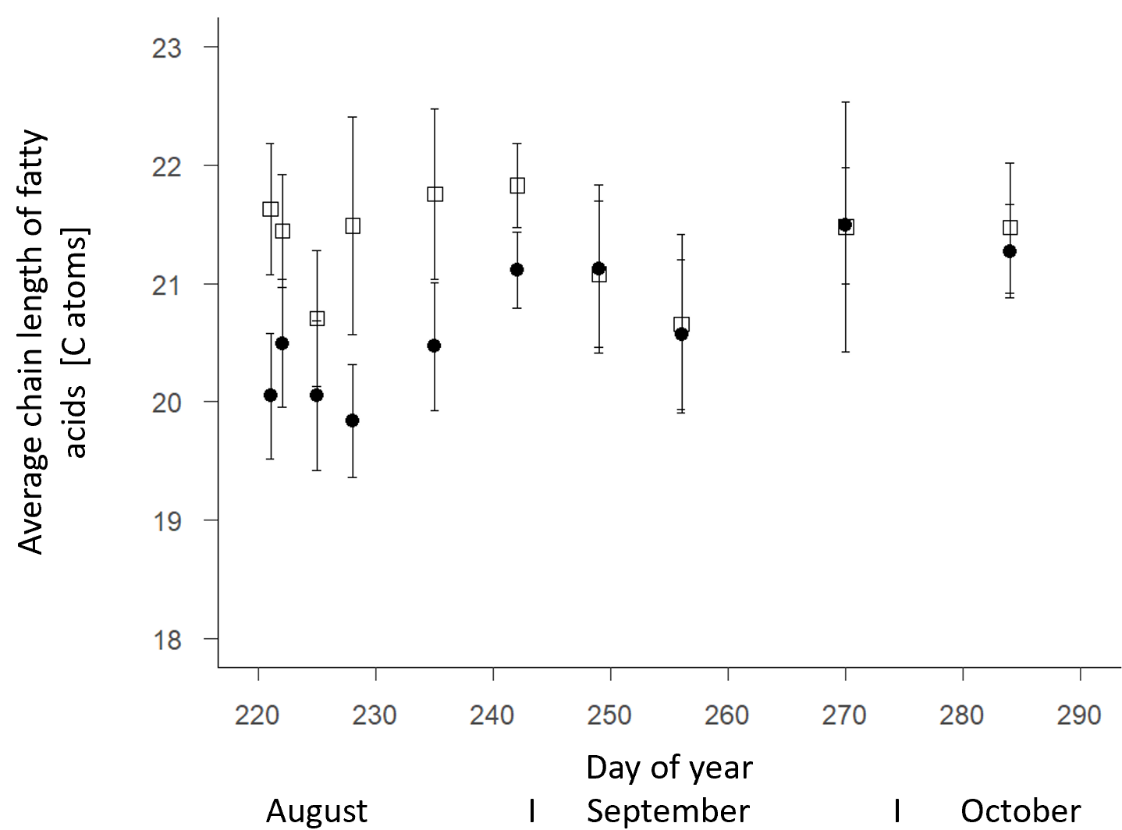


a)

**Supplementary Figure 6** Average chain length of fatty acids (a) and *n*-alkanes (b) of sun-exposed and shaded leaves (field replicates n=4, error bars indicate SE).


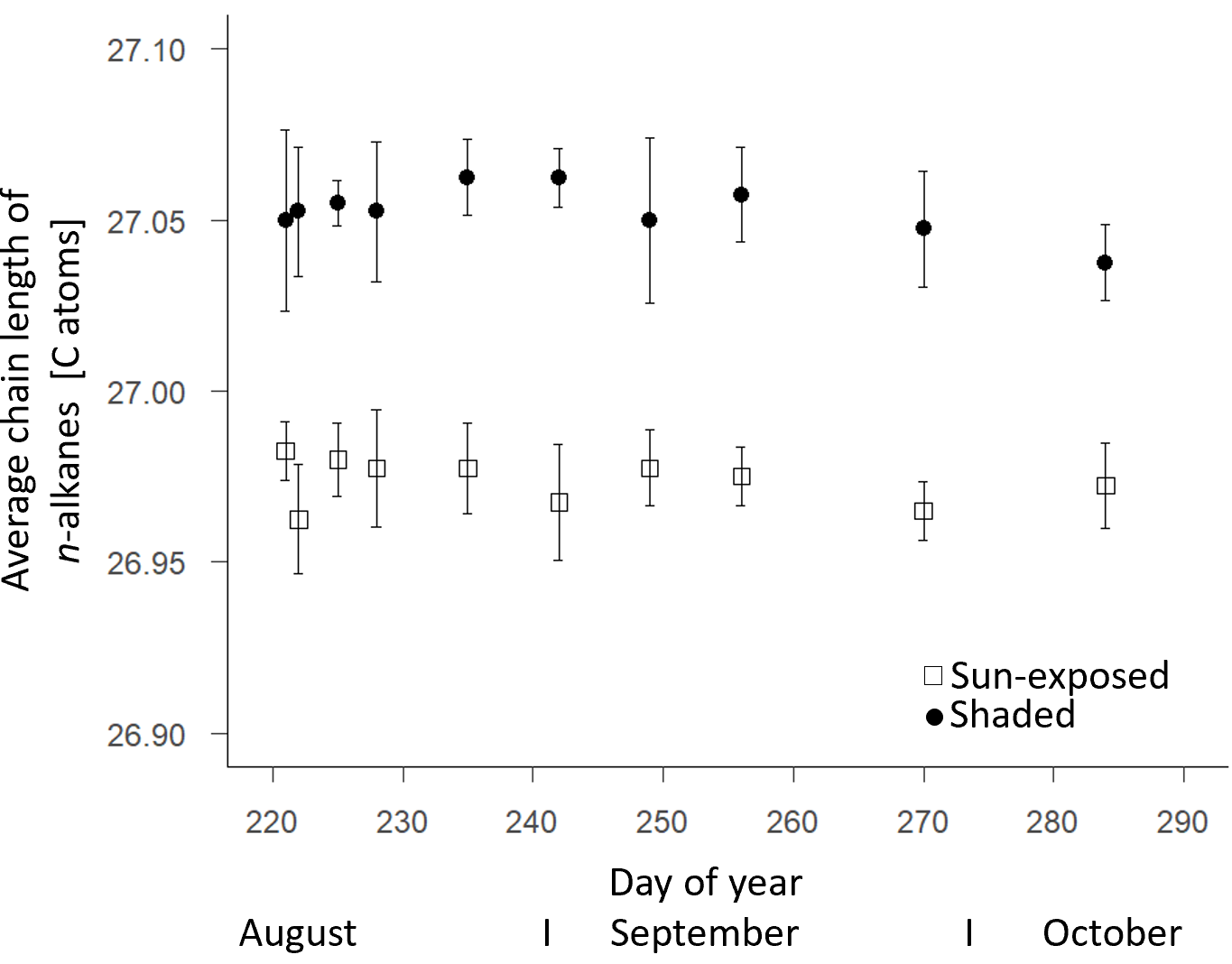


b)


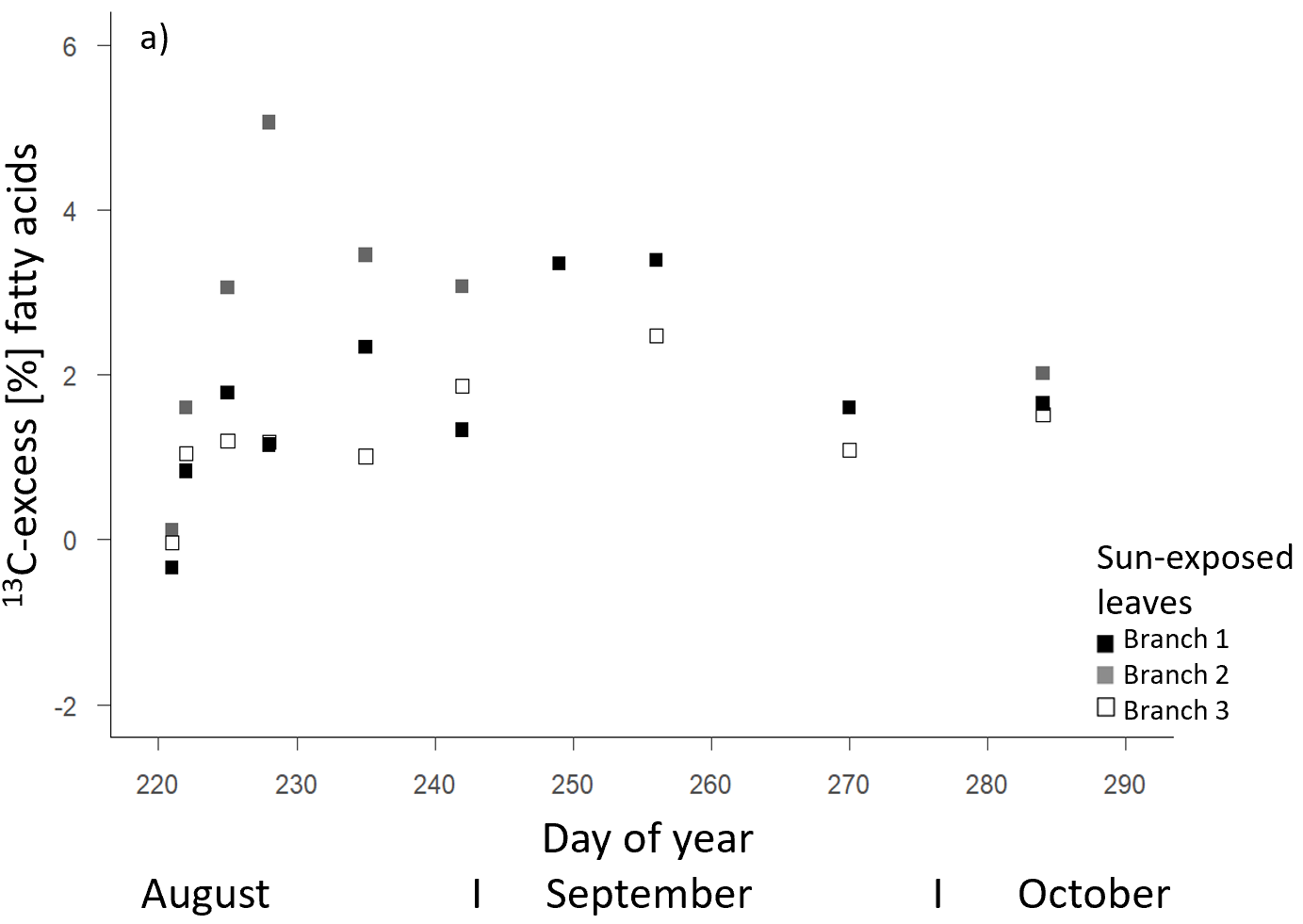

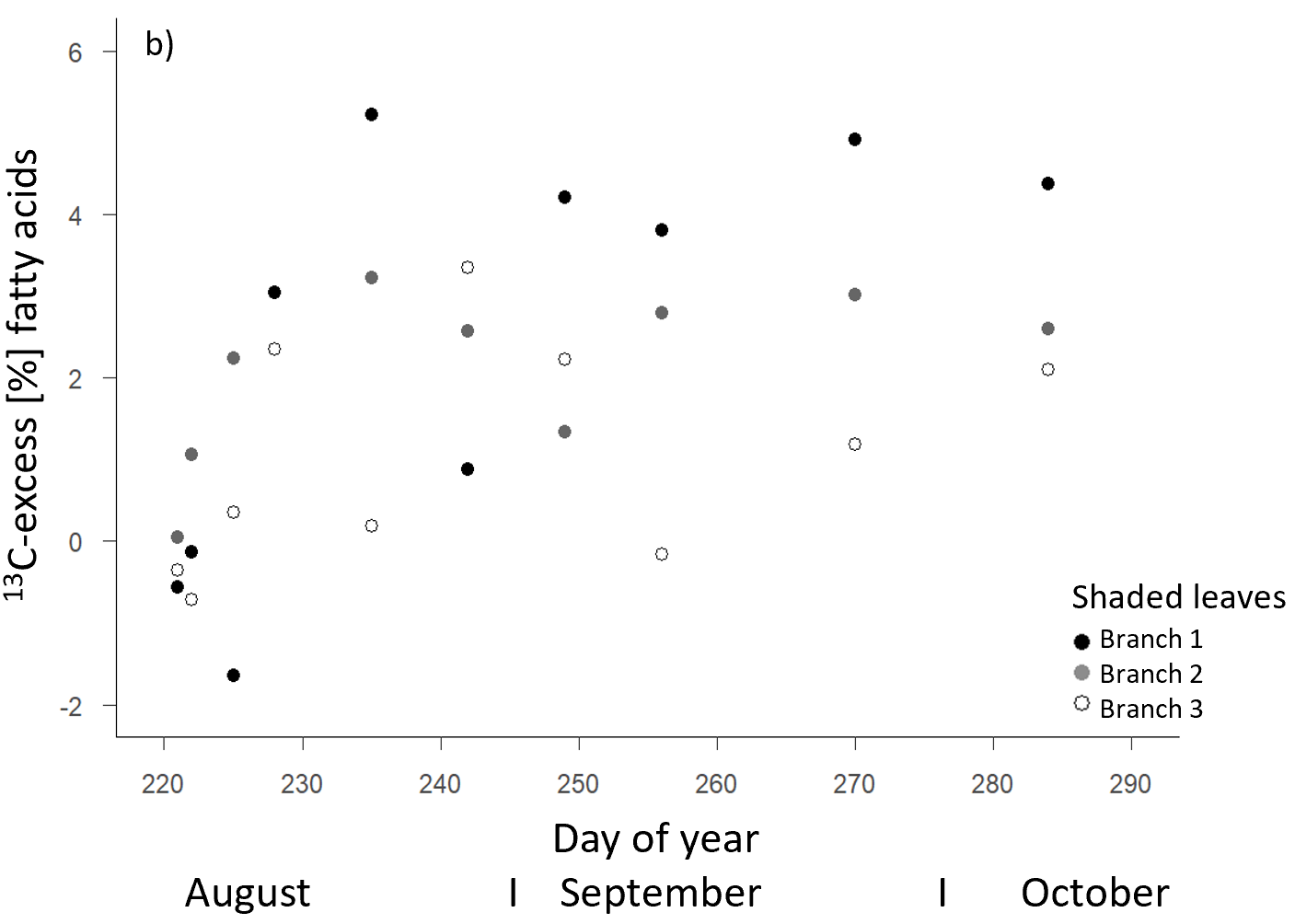

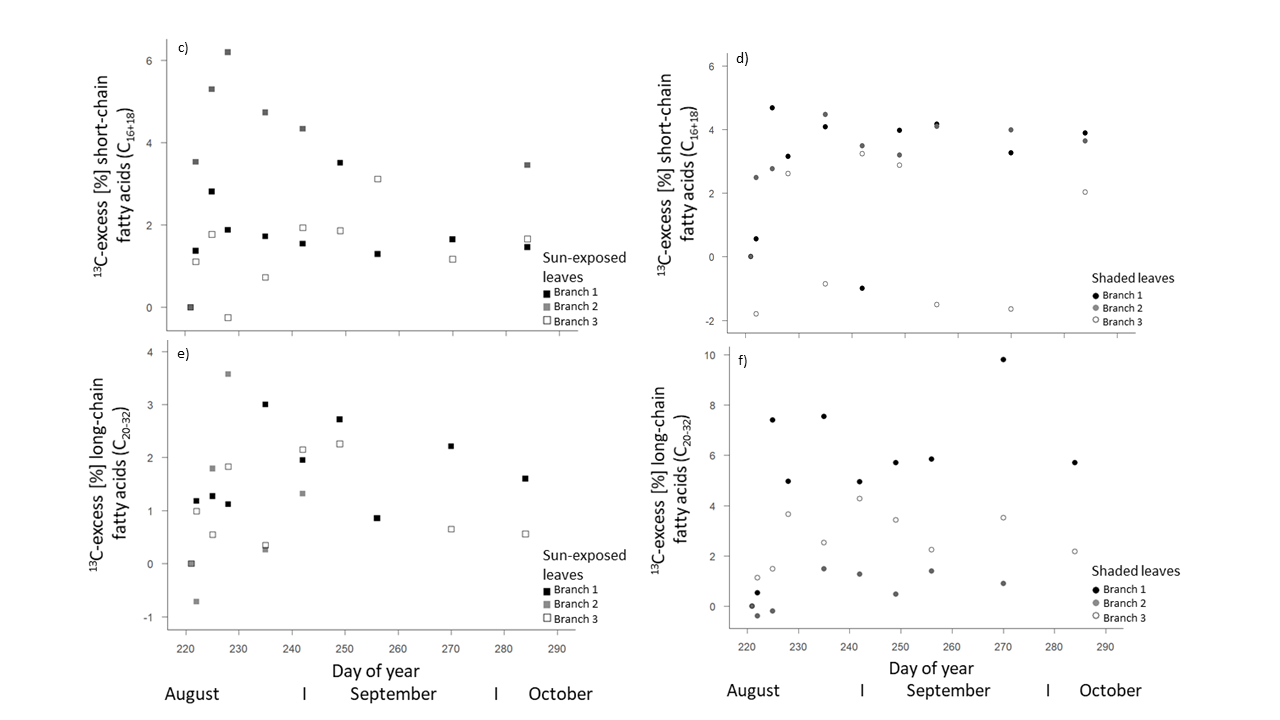


**Supplementary Figure 7** ^13^C-excess of fatty acids of sun-exposed (a) and shaded (a) leaves of individual branches. ^13^C-excess of short-chain (C_16 +18_) fatty acids of sun-exposed (c) and shaded leaves (d) of individual branches. ^13^C-excess of long-chain (C_20-32_) fatty acids of sun-exposed (e) and shaded leaves (f) of individual branches.


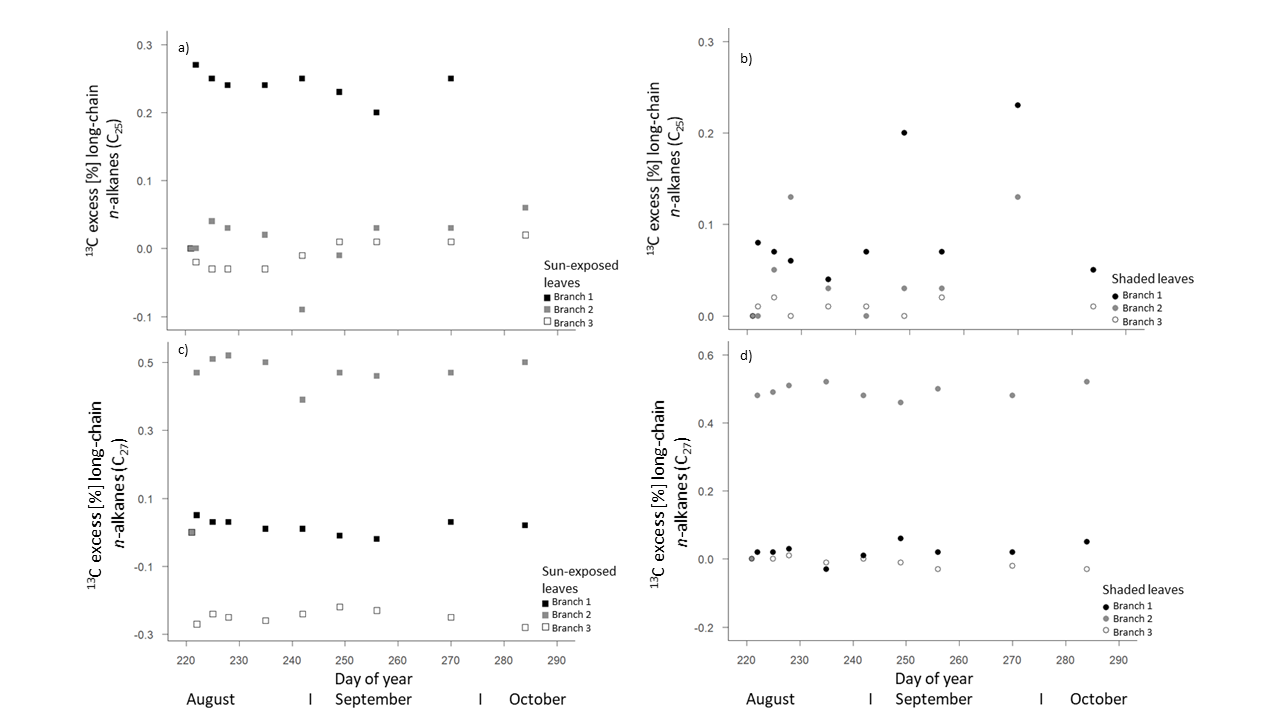


**Supplementary Figure 8** ^13^C-excess of *n*-C_25_ alkane of sun-exposed (a) and shaded leaves (b) of individual branch. ^13^C-excess of *n*-C_27_ alkane of sun-exposed (c) and shaded leaves (d). ^13^C-excess of *n*-C_29_ alkane of sun-exposed (e) and shaded leaves (f).


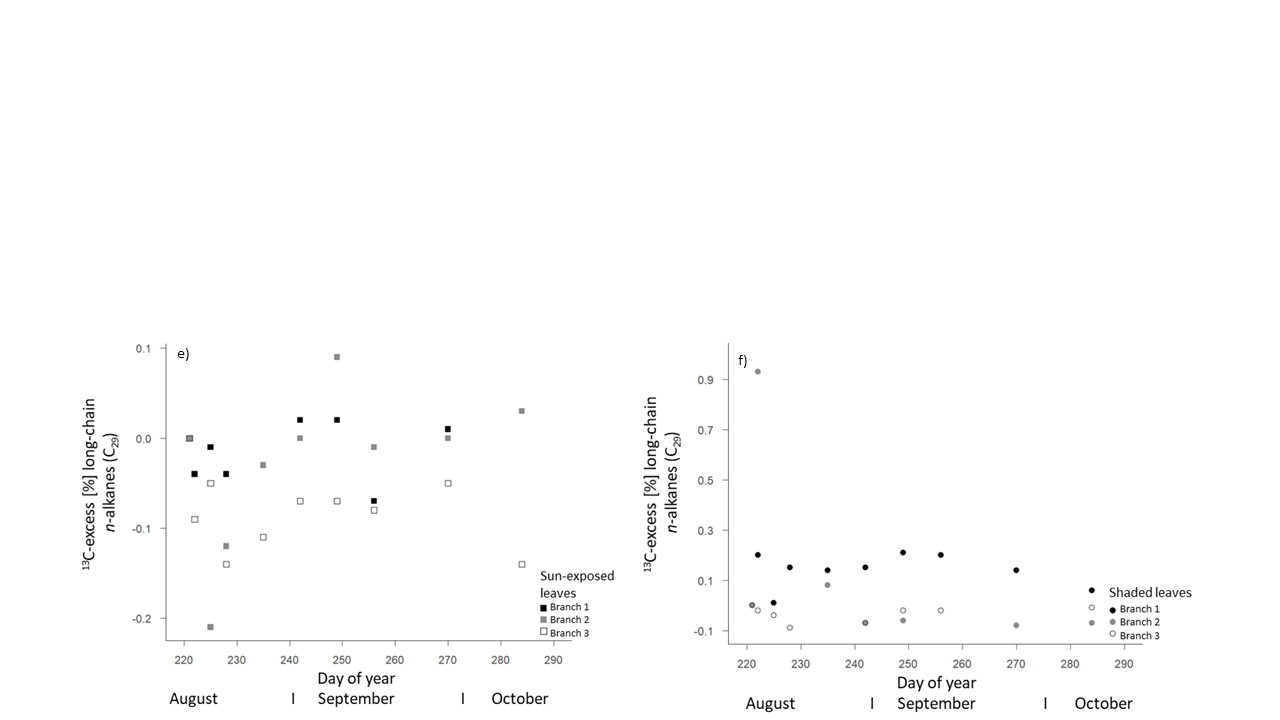

Supplement: Supplementary file 1 [file DataSheet_1.docx]
